# Attitudes and Behaviours Regarding COVID‐19 Mitigation Strategies in Australians With an Underlying Health Condition: A Cross‐Sectional Study

**DOI:** 10.1111/hex.70025

**Published:** 2024-09-12

**Authors:** Sze‐Ee Soh, Darshini Ayton, Amelia Bevins, Helen Skouteris, Mallory Trent, Raina MacIntyre

**Affiliations:** ^1^ Department of Physiotherapy and Rehabilitation, Ageing and Independent Living Research Centre School of Primary and Allied Health Care, Monash University Frankston Victoria Australia; ^2^ Health and Social Care Unit School of Public Health and Preventive Medicine, Monash University Frankston Victoria Australia; ^3^ Biosecurity Program The Kirby Institute, The University of New South Wales Kensington New South Wales Australia; ^4^ College of Public Service & Community Solutions, and College of Health Solutions, Arizona State University Tempe Arizona USA

**Keywords:** attitudes, Australia, behaviours, COVID‐19, health condition, mitigation strategies

## Abstract

**Background:**

Public health strategies have focused on preventing and slowing the transmission of COVID‐19 by promoting the uptake of mitigation strategies. However, little is known about the uptake of these strategies in the presence of underlying health conditions.

**Objectives:**

To describe the attitudes and behaviours of a sample of Australians towards COVID‐19 mitigation strategies, and determine if uptake of these strategies differed across different health conditions.

**Design:**

Cross‐sectional study.

**Setting and Participants:**

National survey of Australian residents over 18 years.

**Main Outcome Measures:**

A purpose‐built survey was used to collect participants' attitudes and behaviours towards COVID‐19 mitigation strategies.

**Results:**

Over half (53%) of the 2867 participants (99% completion rate) reported having one or more comorbidities. The most commonly self‐reported health condition was cardiometabolic conditions (28%). Most participants disagreed that masks were no longer needed (74%) and wanted the 5‐day isolation mandate (66%). More than one‐third would like masks to be mandated for indoor spaces (38%) and 25% avoided going to hospitals. Participants with allergies (OR 1.37; 95% CI 1.14, 1.65), cardiometabolic (OR 1.49; 95% CI 1.23, 1.79), respiratory (OR 1.32; 95% CI 1.07, 1.62) and neurological (OR 1.62; 95% CI 1.12, 2.32) conditions were more likely to avoid using public transport compared to those without. In contrast, participants with underlying mental health conditions were less likely to use N95/P2 facemasks in public spaces (OR 0.46; 95% CI 0.25, 0.87) compared to those without.

**Conclusions:**

A substantial proportion of Australians continued to adopt COVID‐19 mitigation measures or expressed a desire for more mitigations, including mandatory isolation for COVID‐19, despite the lack of mandates. People with an underlying health condition who represent more than half of all adults appear to be more careful with mitigations to avoid COVID‐19.

**Patient or Public Contribution:**

Members of the public were invited to participate in a soft launch of the survey between 4th and 5th January 2023 to test flow and functionality, and to allow the final wording of survey questions to be refined as required.

## 1 Introduction

COVID‐19 was declared a pandemic by the World Health Organisation on 11 March 2020 [1] and ended in May 2023 [1, 2]. Since the advent of COVID‐19, which is caused by the severe acute respiratory syndrome coronavirus 2 (SARS‐CoV‐2), the global number of confirmed cases has exceeded 770 million, and almost 7 million lives have been lost [3].

Until vaccines were available, public health strategies have predominantly focused on preventing and slowing the transmission of the virus by promoting the uptake and adherence to mitigation strategies, such as social distancing and avoidance of crowds, mask wearing, handwashing and sanitiser use [4]. For example, mandates for masks were used in 2020 and 2021 [5, 6], and people who tested positive for COVID‐19 were required to isolate for at least 5 days until late 2021. Studies have shown varying levels of uptake of these strategies within the community [7, 8], which is unsurprising given that behaviour can be influenced by a wide range of factors, including social, cultural, personal and educational factors [9].

Health experts (e.g., World Health Organisation, Australian Chief Medical Officer) warned that people with underlying health conditions, such as cardiovascular disease, diabetes, chronic respiratory disease and conditions, that affect the immune system, would be disproportionately impacted by COVID‐19 in terms of severity of disease and long‐term outcomes [10, 11, 12]. With the achievement of high vaccination rates, public health measures to prevent COVID‐19 infections have eased in Australia. Masks are no longer mandated, even in healthcare settings, and mandatory isolation periods for COVID‐19 ceased in October 2022, although mitigation measures for those at risk of severe illness are still recommended [13]. In contrast, in the United States, 5 days of isolation remained until March 2024, and 1 day of isolation still continues to be recommended [14].

Despite estimates that 47% of Australians are living with one or more chronic conditions, including diabetes, mental conditions and asthma [15], few studies have examined the uptake of mitigation activities in the presence of these underlying health conditions. This present study aims to describe the attitudes and behaviours of a representative sample of Australians towards COVID‐19 mitigation strategies (mask wearing, avoidance of crowds, handwashing/sanitiser, vaccination) in early 2023, and identify the differences in their uptake in relation to the presence of underlying cardiometabolic, respiratory, neurological, immune‐related, allergies and mental health conditions.

## 2 Methods

### 2.1 Study Design and Participants

A national cross‐sectional study of Australian residents aged over 18, carried out by researchers from the NHMRC Centre for Research Excellence (CRE) in Airborne Threats to Health (BREATHE) was conducted. Eligible participants were identified using a global market research company with experience in conducting population surveys for medical research (Dynata) [6]. The survey link was randomly distributed among a geographically targeted sample who met the eligibility criteria of being over 18 years of age and lived in an Australian state or territory. A verification process that included screening questions, participation limits and digital fingerprinting was in place to ensure that responses received were reliable and accurate and to avoid duplicate participants. Data provided were also examined regularly to capture and remove participants with illogical responses, as well as those who did not spend sufficient time on the survey questions.

The sample size for the survey was calculated to identify the proportion of adults that regularly wear facemasks in each Australian state or Territory. Although facemask use in Australia was estimated to be 78% in April 2022 [16], mask wearing has varied depending on fluctuations in COVID‐19 case numbers, mask recommendations and mandates. An estimate of 50% was therefore used for the sample size calculation, where the final sample was determined to be 3073 participants (384 from each state or Territory), which included a 5% margin of error, 80% power and a design effect to account for random sampling.

### 2.2 Survey Instrument

A purpose‐built online survey adapted from a previous survey developed by the research team [6, 17] was used. The survey was part of a larger study of population mask use and risk mitigation behaviours [6, 17]. All survey questions and response options were written by authors (R. M., D. A., H. S., M. T.). The survey was reviewed by senior personnel from the health departments of Victoria and New South Wales (NSW), and input was provided from the respective states. The final survey included a combination of multiple response options and open‐ended questions that consisted of 13 main sections with built‐in skip logic. For the purposes of this study, the sections that were used were eligibility screening (3 questions), demographics (13 questions), mask wearing (4 questions), avoidance of crowds, social distancing and ventilation (14 questions), handwashing and sanitiser use (3 questions) and vaccination (1 question). These sections did not include any open‐ended questions.

### 2.3 Data Collection

Survey responses were collected between 9th and 25th January 2023 and administered using the secure Qualtrics programme, licensed to Monash University. Participants who responded to the Dynata invitation were given access to the survey, which asked a series of screening questions to determine eligibility. Those who responded negatively to one or more of the screening questions were excluded from the survey analysis. Participants who responded affirmatively were directed to an introductory page that included the participant information sheet and detailed explanatory statement. Participants were asked to declare understanding and consent, and were informed on the introductory page that their continued participation in the survey implied consent to the study.

### 2.4 Attitudes and Behaviours Towards COVID‐19 Mitigation Strategies

The main outcomes of interest for this study were participants' attitudes and behaviours towards COVID‐19 mitigation strategies. Their attitudes and behaviours were measured using purposed‐designed questions incorporated within the survey instrument where they responded (agree or disagree) to questions related to mask wearing, avoidance of crowds, social distancing and ventilation, handwashing and sanitiser use, and vaccination (see Appendix S1A).

### 2.5 Underlying Health Conditions

The main exposure for this analysis was participants' self‐report of an underlying health condition. Participants selected the conditions that they have been diagnosed with which were classified into six categories: (1) cardiometabolic (e.g., diabetes, hypertension, heart disease, liver disease, kidney disease); (2) respiratory (e.g., asthma, chronic lung disease, emphysema, chronic bronchitis); (3) immune‐related (e.g., autoimmune conditions, cancer drugs, organ transplantation); (4) allergies (e.g., dermatitis, eczema); (5) neurological (e.g., stroke, epilepsy, Parkinson's disease, acquired brain injury, dementia, migraines); and (6) mental health (e.g., anxiety, depression, post‐traumatic stress disorders, bipolar disorders).

### 2.6 Statistical Analysis

All data analyses were performed using Stata/SE18.0 (StataCorp College Station, Texas, USA). Descriptive statistics were used to summarise participant characteristics including age group (< 45 and ≥ 45 years), gender, Australian state or territory, country of birth, language spoken at home, level of education, living status and self‐reported underlying health condition. The attitudes and behaviours of participants towards COVID‐19 mitigation strategies were also summarised descriptively.

Univariable logistic regression models were used to determine the socio‐demographic characteristics associated with the six self‐reported underlying health conditions. In these models, each underlying health condition was considered to be the dependent variable, while age group, gender, level of education, Australian state or territory and employment status were included as independent variables. Multivariable logistic regression models were subsequently used to examine the association between age group (< 45 vs. ≥ 45 years) and presence of comorbidities (none vs. ≥ 1 comorbidity) with the main outcome of interest, that is, participants' attitudes and behaviours towards COVID‐19 mitigation strategies. These models where age group and presence of co‐morbidities were included as independent variables, were adjusted for the socio‐demographic characteristics that were significantly associated with an underlying health condition.

Multivariable logistic regression models were also used to examine the associations between each underlying health condition as potential predictors of participants' attitudes and behaviours towards COVID‐19 mitigation strategies (i.e., dependent variable). All predictor variables were entered simultaneously into the multivariable models and adjusted for any socio‐demographic characteristics that were significantly associated with an underlying health condition. Highly correlated variables were identified using the variance inflation factor (VIF) where values > 3 were indicative of multicollinearity. Model findings were reported as odds ratio (OR) with 95% confidence intervals (CIs), and *p* < 0.05 was considered to be significant.

## 3 Results

### 3.1 Survey Responses

After excluding responses that were collected during the soft launch (*n* = 169), 2909 participants commenced the survey (Appendix S1B). Of these, 42 did not meet the eligibility criteria resulting in 2867 participants finishing the survey (99% completion rate). Not all participants completed all survey items due to the inclusion of skip logics.

### 3.2 Participant Characteristics

The mean age of participants was 47 years (SD 18), and 54% (*n* = 1556) identified as a woman (Table 1). Participants came from all eight Australian states and territories, and 126 (4%) identified as Aboriginal and Torres Strait Islander. The majority of participants were born in Australia (*n* = 2136; 75%) and spoke English at home (*n* = 2425; 85%). During the survey period, 18% (*n* = 509) lived alone and 53% (*n* = 1523) reported having one or more co‐morbidities, of which 41% were < 45 years of age. The most commonly reported health condition was cardiometabolic conditions (*n* = 808; 28%), followed by allergies (*n* = 662; 23%).

**Table 1:** Socio‐demographic characteristics of participants including self‐reported underlying health conditions.

Characteristic	Participants (*n *= 2867)
Age group (years)	
< 45	1327 (46)
≥ 45	1540 (54)
Gender*	
Woman	1556 (54)
Man	1128 (39)
Nonbinary	10 (< 1)
I use a different term	7 (< 1)
Prefer not to answer	10 (< 1)
State or territory	
New South Wales	414 (14)
Victoria	401 (14)
Queensland	414 (14)
Western Australia	439 (15)
South Australia	428 (15)
Tasmania	271 (9)
Northern Territory	143 (5)
Australian Capital Territory	357 (12)
Country of birth*	
Australia	2136 (75)
Other	565 (20)
Language spoken at home*	
English	2425 (85)
Other	286 (10)
Level of education*	
Primary or elementary school	35 (1)
Secondary or high school	792 (28)
Trade or TAFE qualification	880 (31)
Undergraduate degree	693 (24)
Postgraduate degree	311 (11)
Employment status*	
Employed	1632 (57)
Not employed	1079 (38)
Living status*	
Alone	509 (18)
Not alone	2159 (75)
Health conditions	
Cardiometabolic	808 (28)
Respiratory	500 (17)
Immune‐related	269 (9)
Allergies	662 (23)
Neurological	155 (5)
Mental health	75 (3)

*Note:* All data presented as *n*(%) unless stated otherwise.

* Not all cells sum to 100% owing to missing data.

When we examined the socio‐demographic characteristics of participants across the underlying health conditions, those who were older were more likely to report having underlying cardiometabolic, respiratory, immune‐related and neurological conditions (Appendix S1C). Participants who were not employed were also more likely to report any underlying health condition compared to those who were employed. They had a higher likelihood of reporting cardiometabolic (OR 2.82; 95% CI 2.38, 3.35), respiratory (OR 1.49; 95% CI 1.23, 1.82), immune‐related (OR 3.41; 95% CI 2.61, 4.45), allergies (OR 1.23; 95% CI 1.03, 1.47), neurological (OR 3.75; 95% CI 2.64, 5.33) and mental health conditions (OR 1.96; 95% CI 1.24, 3.12).

### 3.3 COVID‐19 Mitigation Strategies

**Figure 1:**
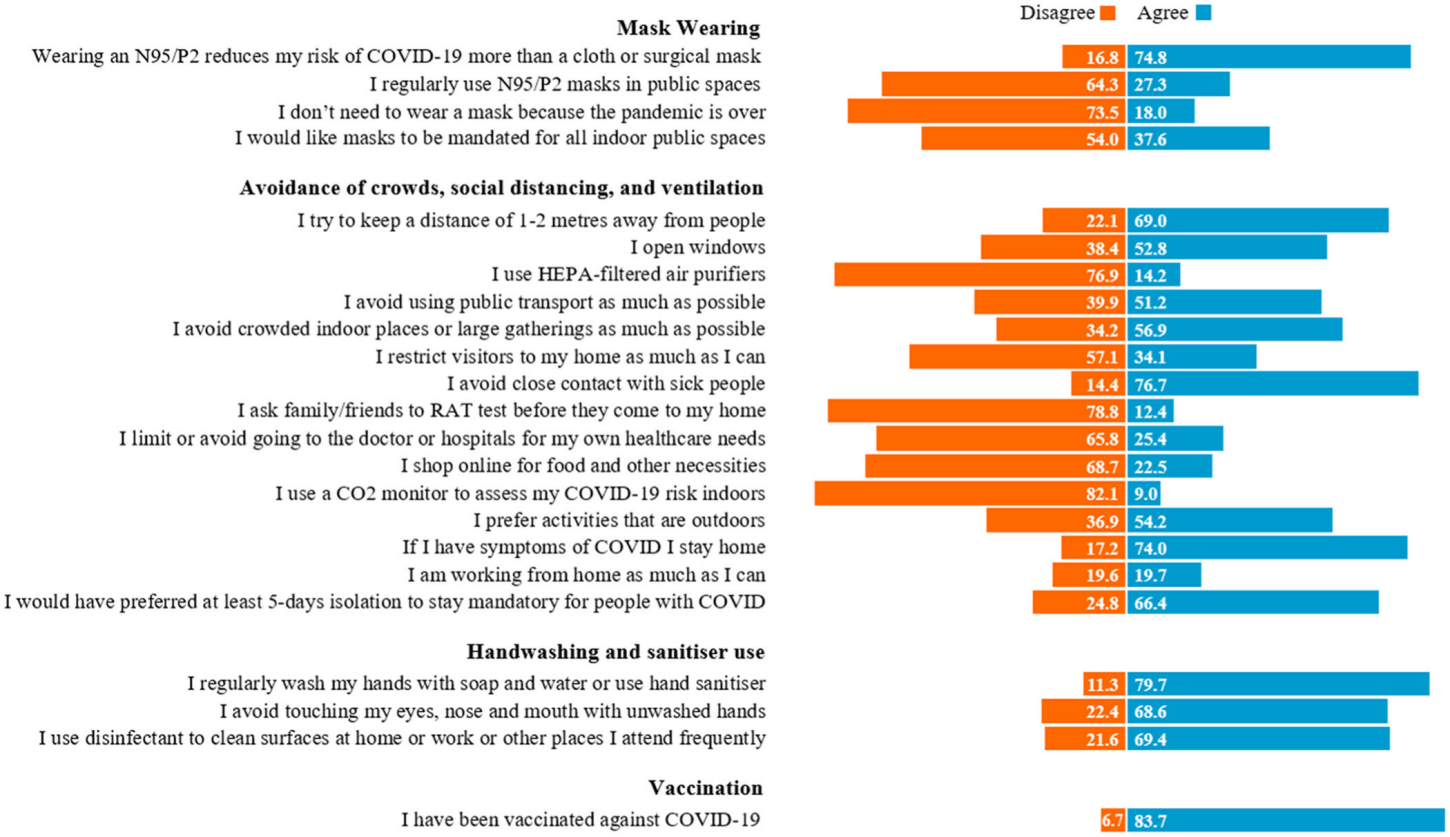
Distribution of participants' attitudes and behaviours towards COVID‐19 mitigation strategies, all data reported as percentages; *not all percentages sum to 100% owing to missing data.

The COVID‐19 mitigation strategies that were adopted by this sample of Australians are shown in Figure 1. Key strategies adopted were being vaccinated against COVID‐19 (*n* = 2400; 84%), regular handwashing (*n* = 2285; 80%) and avoiding close contact with sick people (*n* = 2199; 77%). A majority of participants reported that they felt wearing an N95/P2 face mask reduced their risk of COVID more than a cloth or surgical mask (*n* = 2143; 75%), and that they would stay home if they exhibited symptoms of COVID (*n* = 2121; 74%). Most participants disagreed with the statement that there was no need to wear a mask because the pandemic is over (*n* = 2108; 74%) and would have preferred the 5‐day isolation mandate to remain (*n* = 1903; 66%). However, more than one‐third of respondents would like masks to be mandated for all indoor public spaces (*n* = 1078; 38%), a quarter avoided going to hospitals (*n* = 727; 25%) and a fifth worked from home as much as they could (*n* = 565; 20%).

The adoption of COVID‐19 mitigation strategies varied according to age group and the presence of comorbidities (Appendix S1D). In the multivariable logistic regression model, multicollinearity was not observed between the potential socio‐demographic characteristics associated with an underlying health condition (VIF values < 3 for all variables). As shown in Table 2, participants ≥ 45 years were more likely to adopt COVID‐19 mitigation measures including being vaccinated (OR 2.38; 95% CI 1.73, 3.27), and avoiding high‐risk activities such as close contact with sick people (OR 1.93; 95% CI 1.53, 2.44) and crowded indoor places (OR 1.69; 95% CI 1.42, 2.01) compared to those < 45 years. This group was also more likely to favour mandates for masks indoors (OR 1.32; 95% CI 1.11, 1.57) and 5‐day isolation periods (OR 1.36; 95% CI 1.12, 1.65). In contrast, after adjusting for key socio‐demographic characteristics, there was some variability in the uptake of COVID‐19 mitigation strategies among participants with one or more comorbidities (Table 2).

**Table 2:** Association between age group and presence of comorbidities with participants' attitudes and behaviours towards COVID‐19 mitigation strategies.

	≥ 45 years of age* (*n *= 1540)	≥ 1 comorbidity** (*n *= 1523)
Mask wearing		
N95/P2 better than surgical/cloth	**1.70 (1.36, 2.12)**	**1.42 (1.15, 1.76)**
Regular use of N95/P2 in public	1.06 (0.88, 1.27)	0.90 (0.75, 1.07)
No need for facemasks	**0.36 (0.29, 0.46)**	**0.68 (0.56, 0.83)**
Masks to be mandated indoors	**1.32 (1.11, 1.57)**	1.18 (1.00, 1.39)
Avoidance of crowds, social distancing		
Keep 1–2 m away from people	**1.64 (1.35, 2.00)**	**1.24 (1.03, 1.50)**
Open windows	**1.47 (1.23, 1.74)**	0.95 (0.81, 1.12)
Use HEPA‐filtered air purifiers	**0.44 (0.35, 0.56)**	0.99 (0.79, 1.24)
Avoid public transport	**1.28 (1.08, 1.52)**	**1.32 (1.12, 1.56)**
Avoid crowded indoor places	**1.69 (1.42, 2.01)**	**1.24 (1.05, 1.47)**
Restrict visitors	0.90 (0.75, 1.07)	1.10 (0.93, 1.30)
Avoid close contact with sick people	**1.93 (1.53, 2.44)**	**1.61 (1.29, 2.00)**
Ask family/friends to RAT test	**0.42 (0.33, 0.54)**	0.94 (0.75, 1.19)
Avoid going to the doctor/hospitals	**0.72 (0.60, 0.87)**	0.95 (0.79, 1.14)
Shop online for food	**0.43 (0.35, 0.53)**	1.11 (0.92, 1.35)
Use a CO_2_ monitor	**0.29 (0.21, 0.40)**	0.85 (0.65, 1.11)
Prefer outdoor activities	**1.84 (1.54, 2.19)**	1.10 (0.93, 1.30)
Stay at home if symptomatic	**1.82 (1.46, 2.26)**	1.18 (0.96, 1.45)
Work from home	**0.77 (0.60, 0.99)**	1.07 (0.84, 1.37)
Five‐day isolation to be mandated	**1.36 (1.12, 1.65)**	**1.53 (1.28, 1.84)**
Handwashing and sanitiser use		
Regular hand washing	**1.42 (1.10, 1.85)**	**1.40 (1.10, 1.80)**
Avoid touching eyes, nose and mouth	1.10 (0.90, 1.34)	1.03 (0.86, 1.25)
Use disinfectant to clean	**0.77 (0.63, 0.95)**	**1.25 (1.03, 1.51)**
Vaccination		
Vaccinated against COVID‐19	**2.38 (1.73, 3.27)**	1.21 (0.90, 1.63)

*Note:* All data reported as adjusted odds ratio (OR), 95% CI. Bolded values *p* < 0.05.

* Model adjusted for gender, educational level and employment status as described in Table 1.

** Model adjusted for age, gender, educational level and employment status as described in Table 1.

### 3.4 Association Between Underlying Health Conditions and COVID‐19 Mitigation Strategies

After controlling for key socio‐economic characteristics (i.e., age, gender, educational level and employment status), participants with cardiometabolic conditions were more likely to adopt COVID‐19 mitigation strategies compared to those without (Table 3). In particular, they were more likely to report being vaccinated against COVID‐19 (OR 1.50; 95% CI 1.03, 2.19), shop online for food and other necessities (OR 1.77; 95% CI 1.41, 2.21), use a CO_2_ monitor to assess their risk of COVID‐19 indoors (OR 1.67; 95% CI 1.21, 2.31) and felt that masks should have been mandated for all indoor public spaces (OR 1.60; 95% CI 1.33, 1.92). Participants with cardiometabolic (OR 1.48; 95% CI 1.19, 1.84), respiratory (OR 1.36; 95% CI 1.07, 1.73), immune‐related (OR 1.48; 95% CI 1.06, 2.08) and neurological conditions (OR 2.11; 95% CI 1.31, 3.41) were also more likely to favour a 5‐day isolation period to remain mandatory for people who test positive to COVID‐19. In contrast, participants who reported having an underlying mental health condition were less likely to report that they were regularly using N95/P2 facemasks in public spaces (OR 0.46; 95% CI 0.25, 0.87), although they were more likely to keep 1–2 m away from people (OR 2.27; 95% CI 1.11, 4.65). Multicollinearity was also not observed in these models with VIF values < 3 for all variables.

**Table 3:** Association between each underlying health condition with participants' attitudes and behaviours towards COVID‐19 mitigation strategies.*

	Cardiometabolic conditions (*n *= 808)	Respiratory conditions (*n *= 500)	Immune‐related conditions (*n *= 269)	Allergies (*n *= 662)	Neurological conditions (*n *= 155)	Mental health conditions (*n *= 75)
Mask wearing						
N95/P2 better than surgical/cloth	1.24 (0.97, 1.59)	1.18 (0.90, 1.54)	1.39 (0.94, 2.06)	1.45 (1.13, 1.87)	1.23 (0.77, 1.96)	1.11(0.60, 2.04)
Regular use of N95/P2 in public	**1.32 (1.08, 1.60)**	1.07 (0.86, 1.33)	1.05 (0.79, 1.41)	0.94 (0.77, 1.14)	0.88 (0.60, 1.29)	**0.46 (0.25, 0.87)**
No need for facemasks	0.78 (0.60, 1.00)	0.82 (0.62, 1.07)	0.71 (0.47, 1.07)	**0.78 (0.61, 0.99)**	0.71 (0.43, 1.17)	0.88 (0.47, 1.65)
Masks to be mandated indoors	**1.60 (1.33, 1.92)**	1.14 (0.93, 1.40)	**1.31 (1.00, 1.72)**	1.04 (0.87, 1.25)	1.10 (0.78, 1.54)	0.99 (0.61, 1.60)
Avoidance of crowds, social distancing						
Keep 1–2 m away from people	**1.60 (1.27, 203)**	1.26 (0.98, 1.61)	1.12 (0.79, 1.58)	1.15 (0.93, 1.44)	1.16 (0.74, 1.81)	**2.27 (1.11, 4.65)**
Open windows	1.02 (0.84, 1.23)	1.05 (0.86, 1.29)	1.14 (0.86, 1.50)	0.96 (0.80, 1.15)	0.77 (0.55, 1.09)	0.89 (0.55, 1.43)
Use HEPA‐filtered air purifiers	**1.41 (1.09, 1.84)**	1.13 (0.85, 1.50)	1.29 (0.86, 1.92)	0.79 (0.61, 1.03)	1.32 (0.82, 2.12)	0.69 (0.32, 1.47)
Avoid public transport	**1.49 (1.23, 1.79)**	**1.32 (1.07, 1.62)**	1.30 (0.99, 1.72)	**1.37 (1.14, 1.65)**	**1.62 (1.12, 2.32)**	1.05 (0.65, 1.69)
Avoid crowded indoor places	**1.58 (1.30, 1.93)**	1.08 (0.88, 1.34)	1.33 (0.98, 1.80)	1.14 (0.95, 1.39)	1.49 (0.97, 2.10)	0.96 (0.59, 1.56)
Restrict visitors	**1.39 (1.15, 1.68)**	1.22 (0.99, 1.49)	1.07 (0.82, 1.41)	1.02 (0.85, 1.23)	1.39 (0.99, 1.95)	1.08 (0.67, 1.75)
Avoid close contact with sick people	1.17 (0.89, 1.53)	**1.43 (1.05, 1.95)**	0.96 (0.65, 1.42)	**1.63 (1.24, 2.16)**	1.80 (0.98, 3.32)	1.89 (0.81, 4.44)
Ask family/friends to RAT test	**1.52 (1.16, 2.00)**	1.31 (0.99, 1.74)	1.37 (0.91, 2.06)	**0.68 (0.51, 0.91)**	1.05 (0.63, 1.77)	0.80 (0.39, 1.65)
Avoid going to the doctor/hospitals	1.10 (0.90, 1.36)	1.13 (0.91, 1.41)	1.07 (0.78, 1.46)	0.95 (0.77, 1.16)	1.26 (0.87, 1.81)	1.46 (0.90, 2.38)
Shop online for food	**1.77 (1.41, 2.21)**	1.24 (0.98, 1.56)	1.26 (0.90, 1.76)	0.88 (0.71, 1.09)	1.45 (0.98, 2.14)	0.87 (0.51, 1.51)
Use a CO_2_ monitor	**1.67 (1.21, 2.30)**	1.14 (0.80, 1.61)	0.97 (0.54, 1.74)	**0.59 (0.41, 0.83)**	1.10 (0.58, 2.09)	0.71 (0.28, 1.83)
Prefer outdoor activities	1.16 (0.96, 1.40)	0.94 (0.76, 1.15)	**1.34 (1.00, 1.79)**	1.06 (0.88, 1.27)	1.05 (0.74, 1.50)	0.78 (0.49, 1.26)
Stay at home if symptomatic	1.12 (0.87, 1.43)	1.24 (0.94, 1.63)	1.16 (0.78, 1.71)	1.05 (0.83, 1.33)	1.13 (0.70, 1.85)	1.00 (0.54, 1.87)
Work from home	1.13 (0.84, 1.54)	1.26 (0.91, 1.75)	1.00 (0.58, 1.72)	1.04 (0.77, 1.39)	0.70 (0.36, 1.36)	2.77 (0.85, 9.06)
Five‐day isolation to be mandated	**1.48 (1.19, 1.84)**	**1.36 (1.07, 1.73)**	**1.48 (1.06, 2.08)**	1.23 (0.99, 1.51)	**2.11 (1.31, 3.41)**	1.64 (0.89, 3.04)
Handwashing and sanitiser use						
Regular hand washing	**1.61 (1.19, 2.17)**	0.92 (0.68, 1.26)	1.45 (0.91, 2.30)	1.25 (0.93, 1.68)	1.52 (0.82, 2.82)	0.94 (0.46, 1.94)
Avoid touching eyes, nose and mouth	1.17 (0.94, 1.45)	1.05 (0.83, 1.33)	1.27 (0.91, 1.76)	0.94 (0.76, 1.17)	1.36 (0.88, 2.10)	**0.60 (0.36, 0.99)**
Use disinfectant to clean	1.19 (0.96, 1.48)	**1.33 (1.04, 1.72)**	1.18 (0.86, 1.62)	**1.31 (1.05, 1.64)**	1.49 (0.96, 2.32)	0.76 (0.44, 1.29)
Vaccination						
Vaccinated against COVID‐19	**1.50 (1.03, 2.19)**	0.87 (0.61, 1.25)	1.39 (0.76, 2.54)	1.25 (0.88, 1.79)	1.85 (0.86, 3.97)	1.52 (0.63, 3.65)

*Note:* All data reported as adjusted odds ratio (OR), 95% CI. Bolded values *p* < 0.05.

* Models adjusted for age, gender, educational level and employment status as described in Table 1.

## 4 Discussion

Our findings provide insight into the attitudes and behaviours of a representative sample of Australians towards COVID‐19 mitigation strategies. In particular, we have provided unique data on people with underlying health conditions, which is key because public health rhetoric has repeatedly included ‘protecting the vulnerable’. This may lead to ‘othering’ of people with chronic illness or a perception that they are a minority [18], but our study confirms that half of this sample of Australian adults live with one or more chronic conditions, consistent with previous estimates [15]. Engagement with key strategies including regular handwashing, being vaccinated and avoiding high‐risk activities was relatively high across the sample despite mandates for these measures having ceased. However, the uptake of these strategies varied among those with different underlying health conditions.

Cardiovascular disease is the leading cause of death and disease in Australia and globally, so our finding that people with cardiometabolic conditions are the most likely to use a range of COVID‐19 mitigation measures is important. Cardiometabolic conditions were the most commonly self‐reported health condition, and they were also the group that was most likely to engage in nearly all COVID‐19 mitigation strategies compared to those without. There has been growing evidence that conditions, such as hypertension, heart disease and diabetes, are linked to increased severity of disease and poorer health outcomes including death as a result of COVID‐19 [11, 19, 20]. This has been reflected in the public health messaging in Australia, where early access to vaccines and boosters was provided to individuals with these conditions [21].

Interestingly, participants with respiratory conditions had relatively low uptake of COVID‐19 mitigation strategies despite being at risk of having poor outcomes [10, 12]. Although the use of facemasks has been strongly advocated as a method of reducing transmission, and vaccination has been shown to be effective at reducing the severity of the disease [22, 23], uptake of these strategies in this group was not statistically significant. Given that asthma is one of the most common comorbidities in Australia [24], more work exploring the barriers and enablers towards the adoption of such strategies may be needed in this group of individuals. People with compromised lung function may have difficulties using masks, so tailored strategies to improve safe indoor air with better ventilation and reduce exposure risk may be needed.

Despite variability in the adoption of COVID‐19 mitigation strategies, participants with cardiometabolic, respiratory, immune‐related and neurological health conditions were more likely to favour a mandatory 5‐day isolation period compared to those without the conditions. In contrast, only participants with cardiometabolic and immune‐related conditions favoured facemasks to remain mandatory for indoor public spaces. The preference for mandatory isolation is consistent with the key mitigation strategies that were adopted by this sample of participants, which focused predominantly on avoiding high‐risk activities. It is also consistent with findings from the United States, where the majority of participants with similar underlying health conditions preferred to work from home or stay home if they felt unwell [7]. Given the complexity and misconceptions regarding the use of facemasks [17], a more sustainable strategy to minimise COVID‐19 transmission within the community may be to encourage people to remain at home if they are not feeling well.

Some limitations need to be considered. First, participants were recruited through a panel of Dynata members, which may have introduced a potential selection bias. The sample was over‐represented with Australian‐born, English‐speaking people, so further research is required on migrant communities. The survey may also not have captured the perspectives of individuals who are not familiar with using technology (e.g., online surveys) or have limited access to the internet. Additionally, the data collected may be subject to recall and social desirability bias, given the focus of the survey was on COVID‐19 mitigation strategies. The predominant use of closed‐ended survey questions to measure participants' attitudes and behaviours was another limitation that needs to be considered. Further qualitative research would be helpful to explore and understand attitudes and behaviours towards COVID mitigation strategies. Nevertheless, this was a nationwide survey that included the collection of various socio‐demographic and behaviour variables. As such, given the large sample size and span of demographic characteristics, our findings provide a nationally representative snapshot of the attitudes and behaviours of a sample of Australians towards COVID‐19 mitigation strategies.

Our findings showed that in early 2023 a high proportion of Australians continued to use mitigation measures against COVID‐19, despite the lack of mandates. We also confirmed that people with underlying health conditions are not a minority but comprise half of adults. It is, therefore, important in public health messaging to recognise that chronic health conditions are common in the population and that COVID‐19 may remain a public health threat for a large proportion of the population. Additionally, within this group, we identified that those with cardiometabolic conditions will most likely continue to use mitigation measures against COVID‐19, such as regular mask wearing, while those with respiratory disease may require more tailored protections that take into consideration difficulty with wearing masks. This information can inform public policy during times of high community transmission of COVID‐19 or other respiratory pathogens.

## Author Contributions

**Sze‐Ee Soh:** writing–original draft, methodology, formal analysis, writing–review & editing, investigation. **Darshini Ayton:** conceptualisation, writing–original draft, writing–review & editing, methodology. **Amelia Bevins:** writing–review & editing, writing–original draft, methodology, formal analysis. **Helen Skouteris:** conceptualisation, writing–review & editing, supervision. **Mallory Trent:** conceptualisation, investigation, writing–review & editing. **Raina MacIntyre:** conceptualisation, funding acquisition, investigation, writing–review & editing, project administration, supervision.

## Ethics Statement

Ethics approval was gained from the University of New South Wales Human Research Ethics Committee (approval date: 2 December 2022; ref: HC220737). Participants provided informed consent to participate in the study before completing the survey.

## Conflicts of Interest

The authors declare no conflicts of interest.

## Supporting information

Supporting information.

## Data Availability

The data that support the findings of this study are available on request from the corresponding author. The data are not publicly available due to privacy or ethical considerations.
