# Supplementary material for: Attitudes and Behaviours Regarding COVID‐19 Mitigation Strategies in Australians With an Underlying Health Condition: A Cross‐Sectional Study
Source: Health Expect. 2024 Sep 12;27(5):e70025. doi: 10.1111/hex.70025 (PMC11391943; doi:10.1111/hex.70025)
Supplement: Supplementary file 1 — Supporting information. [file HEX-27-e70025-s001.docx]

**Supplementary Appendix A:** COVID-19 mitigation strategies

Mask wearing

- Wearing an N95/P2 reduces my risk of COVID-19 more than a cloth or surgical mask
- I regularly use N95/P2 masks in public spaces
- I don’t need to wear a mask because the pandemic is over
- I would like masks to be mandated for all indoor public spaces

Avoidance of crowds, social distancing and ventilation

- I try to keep a distance of 1-2 metres away from people to reduce my risk of COVID-19
- I open windows to reduce my risk of COVID-19
- I avoid using public transport as much as possible to reduce my risk of COVID-19
- I avoid crowded indoor places or large gatherings as much as possible to reduce my risk of COVID-19
- I restrict visitors to my home as much as I can to reduce my risk of COVID-19
- I avoid close contact with sick people
- I ask family/friends to RAT test before they come to my home
- I limit or avoid going to the doctor or hospitals for my own healthcare needs because I am worried about catching COVID-19
- I shop online for food and other necessities
- I use a CO_2_ monitor to assess my COVID-19 risk indoors
- I prefer activities that are outdoors because I think it reduces my risk of COVID-19
- If I have symptoms of COVID I stay home
- I am working from home as much as I can to reduce my risk of COVID-19
- I would have preferred at least 5-days isolation to remain mandatory for people with COVID-19

Handwashing and sanitiser use

- I regularly wash my hands with soap and water or use hand sanitiser to reduce my risk of COVID-19
- I avoid touching my eyes, nose and mouth with unwashed hands
- I use disinfectant to clean surfaces at home or work or other places I attend frequently

Vaccination

- I have been vaccinated against COVID-19

**
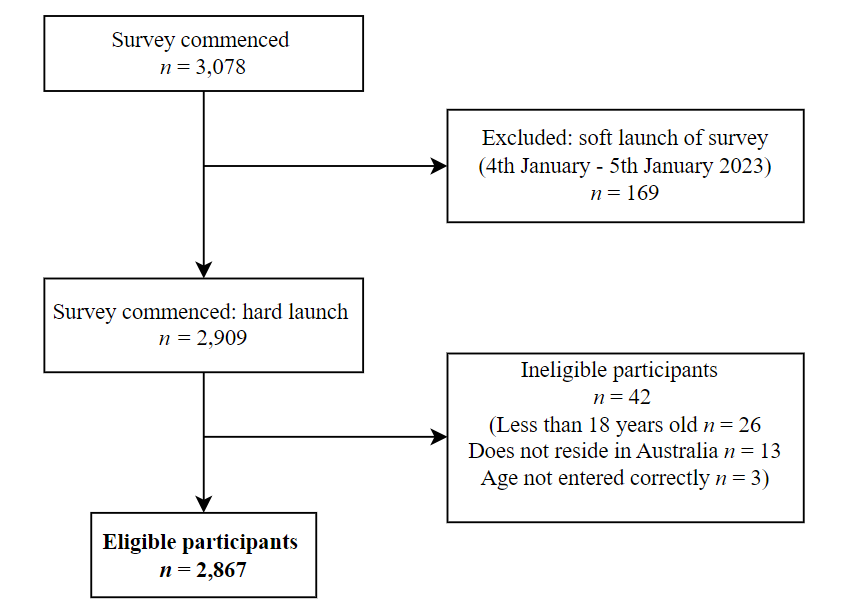
Supplementary Appendix B:** Participant flowchart with exclusion criteria

**Supplementary Appendix C:** Socio-demographic characteristics by underlying health conditions

|  | **Cardiometabolic conditions**  **(*n* = 808)** | **Respiratory conditions**  **(*n* = 500)** | **Immune-related conditions**  **(*n* = 269)** | **Allergies**  **(*n* = 662)** | **Neurological conditions**  **(*n* = 155)** | **Mental health conditions**  **(*n* = 75)** |
| --- | --- | --- | --- | --- | --- | --- |
| Age  <45 years  ≥45 years | 1.0 [reference]  **4.86 (4.02. 5.88)** | 1.0 [reference]  **1.28 (1.06, 1.56)** | 1.0 [reference]  **4.59 (3.32, 6.34)** | 1.0 [reference]  1.00 (0.84, 1.18) | 1.0 [reference]  **1.81 (1.29, 2.55)** | 1.0 [reference]  1.10 (0.69, 1.74) |
| Gender  Woman  Man  Non-binary  I use a different term  Prefer not to answer | 1.0 [reference]  **2.17 (1.84, 2.57)**  0.83 (0.18, 3.94)  #  # | 1.0 [reference]  **0.69 (0.57, 0.85)**  1.64 (0.42, 6.39)  1.53 (0.30, 7.94)  # | 1.0 [reference]  **1.93 (1.50, 2.49)**  #  2.07 (0.25, 17.33)  # | 1.0 [reference]  **0.65 (0.54, 0.78)**  1.74 (0.49, 6.20)  1.04 (0.20, 5.40)  # | 1.0 [reference]  0.78 (0.55, 1.09)  #  2.48 (0.30, 20.80)  # | 1.0 [reference]  **0.57 (0.35. 0.9)**  **7.38 (1.53, 35.62)**  #  # |
| Level of education  Primary or elementary school  Secondary or high school  Trade or TAFE qualification  Undergraduate (Bachelor’s) degree  Postgraduate degree | 1.0 [reference]  0.54 (0.27, 1.07)  0.57 (0.29, 1.12)  0.41 (0.21, 0.82)  0.42 (0.21, 0.85) | 1.0 [reference]  0.93 (0.40, 2.16)  1.06 (0.46, 2.48)  0.75 (0.32, 1.77)  0.75 (0.31, 1.81) | 1.0 [reference]  1.17 (0.35, 3.89)  1.34 (0.40, 4.45)  1.14 (0.34, 3.83)  0.85 (0.24, 3.00) | 1.0 [reference]  0.91 (0.41, 2.04)  1.23 (0.55, 2.74)  1.16 (0.52, 2.60)  1.05 (0.46, 2.42) | 1.0 [reference]  **0.20 (0.08, 0.48)**  **0.29 (0.12, 0.70)**  **0.25 (0.10, 0.60)**  **0.15 (0.05, 0.41)** | 1.0 [reference]  0.33 (0.10, 1.16)  0.38 (0.11, 1.30)  **0.19 (0.05, 0.70)**  **0.21 (0.05, 0.88)** |
| State in Australia  New South Wales  Victoria  Queensland  Western Australia  South Australia  Tasmania  Northern Territory  Australian Capital Territory | 1.0 [reference]  1.15 (0.85, 1.57)  1.09 (0.80, 1.48)  1.10 (0.82, 1.50)  1.44 (1.07, 1.94)  1.38 (0.99, 1.93)  0.67 (0.42, 1.07)  1.00 (0.72, 1.38) | 1.0 [reference]  1.17 (0.79, 1.74)  1.36 (0.92, 1.99)  1.26 (0.86, 1.86)  1.88 (1.30, 2.71)  **2.10 (1.41, 3.14)**  1.21 (0.71, 2.07)  1.48 (1.00, 2.20) | 1.0 [reference]  1.26 (0.76, 2.09)  1.56 (0.96, 2.53)  1.35 (0.83, 2.21)  **1.73 (1.08, 2.78)**  1.24 (0.71, 2.18)  0.66 (0.28, 1.53)  1.35 (0.81, 2.25) | 1.0 [reference]  1.40 (0.99, 2.00)  1.44 (1.02, 2.03)  1.37 (0.97, 1.93)  **1.67 (1.20, 2.34)**  **2.02 (1.40, 2.92)**  1.18 (0.72, 1.91)  **1.65 (1.17, 2.34)** | 1.0 [reference]  1.03 (0.52, 2.05)  1.44 (0.76, 2.72)  1.72 (0.93, 3.16)  1.27 (0.66. 2.42)  1.27 (0.62, 2.63)  0.85 (0.31, 2.34)  1.83 (0.78, 3.44) | 1.0 [reference]  0.87 (0.39, 1.97)  0.69 (0.29, 1.62)  0.79 (0.35, 1.79)  1.12 (0.53, 2.38)  1.43 (0.64, 3.18)  -  0.35 (0.11, 1.08) |
| Employment status  Employed  Not employed | 1.0 [reference]  **2.82 (2.38, 3.35)** | 1.0 [reference]  **1.49 (1.23, 1.82)** | 1.0 [reference]  **3.41 (2.61, 4.45)** | 1.0 [reference]  **1.23 (1.03, 1.47)** | 1.0 [reference]  **3.75 (2.64, 5.33)** | 1.0 [reference]  **1.96 (1.24, 3.12)** |

All data reported as odds ratio (OR), 95% CI

Bolded values *p*<0.05

#Excluded from analysis owing to low cell count

**Supplementary Appendix D:** Adoption of COVID-19 mitigation strategies by age group and presence of comorbidities

|  | **<45 years of age**  **(*n*=1,327)** | | **≥45 years of age**  **(*n*=1,540)** | | **No comorbidity**  **(*n*=1,344)** | | **≥1 comorbidity**  **(*n*=1,523)** | |
| --- | --- | --- | --- | --- | --- | --- | --- | --- |
| Mask wearing  N95/P2 better than surgical/cloth  Regular use of N95/P2 in public  No need for facemasks  Masks to be mandated indoors | 952  352  336  437 | (72)  (27)  (25)  (33) | 1,191  430  181  641 | (77)  (28)  (12)  (42) | 902  356  285  430 | (67)  (26)  (21)  (32) | 1,241  426  232  648 | (81)  (28)  (15)  (43) |
| Avoidance of crowds, social distancing  Keep 1-2m away from people  Open windows  Use HEPA-filtered air purifiers  Avoid public transport  Avoid crowded indoor places  Restrict visitors  Avoid close contact with sick people  Ask family/friends to RAT test  Avoid going to the doctor/hospitals  Shop online for food  Use a CO_2_ monitor  Prefer outdoor activities  Stay at home if symptomatic  Work from home  5-days isolation to be mandated | 833  648  262  629  652  445  954  226  387  402  191  621  913  360  834 | (63)  (49)  (20)  (13)  (47)  (49)  (34)  (17)  (29)  (30)  (14)  (47)  (69)  (27)  (63) | 1,146  865  146  839  980  532  1,245  129  340  242  67  933  1,208  205  1,069 | (74)  (56)  (9)  (54)  (64)  (35)  (81)  (8)  (22)  (16)  (4)  (61)  (78)  (13)  (69) | 814  656  200  584  652  406  905  174  337  294  141  641  890  293  765 | (61)  (49)  (15)  (43)  (49)  (30)  (67)  (13)  (25)  (22)  (10)  (48)  (66)  (22)  (57) | 1,165  857  208  884  980  571  1,294  181  390  350  117  913  1,231  272  1,138 | (76)  (56)  (14)  (58)  (64)  (37)  (85)  (12)  (26)  (23)  (8)  (60)  (81)  (18)  (75) |
| Handwashing and sanitizer use  Regular hand washing  Avoid touching eyes, nose and mouth  Use disinfectant to clean | 1,035  897  964 | (78)  (68)  (73) | 1,250  1,069  1,025 | (81)  (69)  (67) | 972  851  859 | (72)  (63)  (64) | 1,313  1,115  1,130 | (86)  (73)  (97) |
| Vaccination  Vaccinated against COVID-19 | 1,068 | (80) | 1,332 | (86) | 1,034 | (77) | 1,366 | (90) |

Not all cells sum to 100% owing to missing data
